# Characterization of the *Gbx1^−/−^* Mouse Mutant: A Requirement for *Gbx1* in Normal Locomotion and Sensorimotor Circuit Development

**DOI:** 10.1371/journal.pone.0056214

**Published:** 2013-02-13

**Authors:** Desirè M. Buckley, Jessica Burroughs-Garcia, Mark Lewandoski, Samuel T. Waters

**Affiliations:** 1 Division of Biological Sciences, University of Missouri, Columbia, Missouri, United States of America; 2 Christopher S. Bond Life Sciences Center, University of Missouri, Columbia, Missouri, United States of America; 3 Cancer and Developmental Biology Laboratory, National Cancer Institute, National Institutes of Health, Frederick, Maryland, United States of America; Ecole Normale Supérieure de Lyon, France

## Abstract

The Gbx class of homeobox genes encodes DNA binding transcription factors involved in regulation of embryonic central nervous system (CNS) development. *Gbx1* is dynamically expressed within spinal neuron progenitor pools and becomes restricted to the dorsal mantle zone by embryonic day (E) 12.5. Here, we provide the first functional analysis of *Gbx1*. We generated mice containing a conditional *Gbx1* allele in which exon 2 that contains the functional homeodomain is flanked with *lox*P sites (*Gbx1^flox^*); Cre-mediated recombination of this allele results in a *Gbx1* null allele. In contrast to mice homozygous for a loss-of-function allele of *Gbx2*, mice homozygous for the *Gbx1* null allele, *Gbx1^−/−^*, are viable and reproductively competent. However, *Gbx1^−/−^* mice display a gross locomotive defect that specifically affects hindlimb gait. Analysis of embryos homozygous for the *Gbx1* null allele reveals disrupted assembly of the proprioceptive sensorimotor circuit within the spinal cord, and a reduction in ISL1^+^ ventral motor neurons. These data suggest a functional requirement for *Gbx1* in normal development of the neural networks that contribute to locomotion. The generation of this null allele has enabled us to functionally characterize a novel role for *Gbx1* in development of the spinal cord.

## Introduction

The precise assembly of sensorimotor circuits within the spinal cord during development plays a critical role in defining the control of motor behavior in the mature organism. Each neuronal cell type requires molecular mechanisms that establish progenitor pool sizes and subsequently regulate the differentiation of precursors into unique subpopulations of post-mitotic neurons**.** Transcriptional networks exist in the developing hindbrain and spinal cord to control the specification, organization and functional properties of neurons, which contribute to motor control systems [1]–[7]. While many of these developmentally essential genes have been characterized through gene inactivation studies, much work is still needed to elucidate the unique functional capabilities that allow them to contribute to the neural networks that exist within the spinal cord.

Neural circuitry within the spinal cord, a key component to the repertoire of vertebrate motor tasks, is critical in the perception of and reaction to sensory information from the external environment. A characteristic feature of all neuronal transmission within the spinal cord includes the essential involvement of afferent axons, efferent axons, interneurons and motor neurons (MNs). The specific connective arrangement of these important constituents of neural circuitry determines and contributes to the diverse function harbored by neural networks [8], [9]. The afferents of neuronal cell bodies that reside within the dorsal root ganglia (DRG) adjacent to the spinal cord project into the dorsal spinal cord, and establish precise connections with interneurons in their target zones. The correct trajectory and establishment of these connections, is critical for processing sensory information from the periphery. The neural cell bodies in the DRG give rise to three major sensory modalities from a heterogeneous population of multipotent neural crest (NC) cells, distinguished only by their unique gene expression. Sensory neuron subtypes include nociceptors that sense pain and express the neurotrophic tyrosine receptor kinase (Trk) A, mechanoreceptors that sense touch and express TrkB, and proprioceptors that sense spatial anatomical orientation and express TrkC [10], [11]. The type, or modality, of sensory stimuli orchestrated is contingent upon the precise connectivity of sensory neurons with subpopulations of interneurons which reside in distinct laminae of the dorsal horn [12]. Specification and patterning of dorsal sensory interneurons requires the differential expression of basic helix-loop-helix (bHLH) and homeodomain-containing transcription factors in response to BMP/TGF-ß signaling [13]. Development of sensory interneurons within the dorsal spinal cord occurs in two waves of neurogenesis [14], [15]. Sensory interneurons that populate deep layers of the dorsal horn are generated in the early phase, embryonic day (E) 10–E11, from six classes of precursors within the ventricular zone pd1 - pd6 [9], [16]. The interneurons that populate the superficial layers of the dorsal horn arise during the late phase of neurogenesis, E12–E13.5, from the most ventral progenitor zones and ultimately generate either inhibitory GABAergic (dILA), or excitatory glutamatergic (dILB) interneurons [14], [15].

In contrast to the dorsal spinal cord, the ventral spinal cord is patterned by a gradient of Sonic Hedgehog (SHH) protein secreted by the floorplate and notochord, and contains the core of spinal locomotor circuitry. Cells that reside in five ventral progenitor domains (pMN and pV0 – pV3) are influenced by the differential expression of homeodomain genes and generate motor neurons and four classes of interneurons. Each general class of neuron appears to be comprised of cells capable of differentiating into molecularly distinct neuronal subtypes [5], [17], [18]. Interestingly, motor control requires interaction between the dorsal and ventral compartments of the spinal cord. For example, while the initial innervation by proprioceptive afferents occurs in the dorsal spinal cord, the ventral spinal cord contains the neurons that regulate the motor output for this somatosensory circuit including the somatic motor neurons, V1-derived Ia inhibitory neurons and V0 commissural interneurons [9], [19].

The gastrulation brain homeobox (Gbx) class of homeobox genes encode for two DNA binding transcription factors, GBX1 and GBX2. Although *Gbx2^−/−^* mice die at the day of birth, analysis of embryos from several species have provided clear evidence that *Gbx2* is required in establishment of the midbrain-hindbrain boundary as well as patterning and growth along the anteroposterior axis in the hindbrain [20]–[24]. In the zebrafish embryo, *gbx2* knockdown results in a truncation of the hindbrain region between anterior rhombomere (r) 1 and anterior r3. In addition, severe clustering abnormalities of motor neuron (MN) cell bodies within cranial nerve V, a derivative of r2 and r3, occur [24]. Similar developmental defects have been shown within the anterior hindbrain of *Gbx2* deficient mouse embryos, demonstrating an evolutionarily conserved role for *Gbx2* in embryonic development [20], [21]. In addition, recent fate mapping studies in mouse embryos have demonstrated that some ventral motor neurons as well as dorsal and ventral interneurons of the spinal cord are derived from the *Gbx2* lineage [25].

*Gbx1* is dynamically expressed within the developing central nervous system and is detected in the neural plate by E8.25. At E10.5, *Gbx1* mRNA transcripts are broadly detected within the ventricular zone of the spinal cord, and become restricted to the dorsal mantle zone by E12.5 [26], [27]. Upon expression in the dorsal mantle zone, GBX1 protein co-localizes with LBX1^+^, LHX1/5^+^, and PAX2^+^ (class B) GABAergic neurons in the dorsal spinal cord which persists throughout adulthood [27]. While the mechanisms by which *Gbx1* functions as a transcriptional regulator are still to be determined, collectively these data suggest that murine *Gbx1* may be involved in the development of interneurons and motor neurons within the dorsal and ventral spinal cord. To identify the function of *Gbx1* in development of the nervous system, we created a conditional *Gbx1* (floxed) allele that can be converted to a null allele via Cre-mediated recombination [28]. *Gbx1^−/−^* mice exhibit a profound locomotive defect specifically affecting hind-limb gait. Molecular analysis of mutant embryos reveals abnormal projection of proprioceptive sensory afferents into the dorsal spinal cord suggesting a disorganized assembly of proprioceptive sensorimotor circuitry within the spinal cord. Moreover, we show a marked loss of ISL1^+^ ventral MNs beginning at E14.5. Taken together, these data demonstrate that *Gbx1* function contributes to the development of the neural network that contributes to normal locomotion.

## Results

### *Gbx1^−/−^* Mice Exhibit a Profound Locomotive Defect

**Figure 1:**
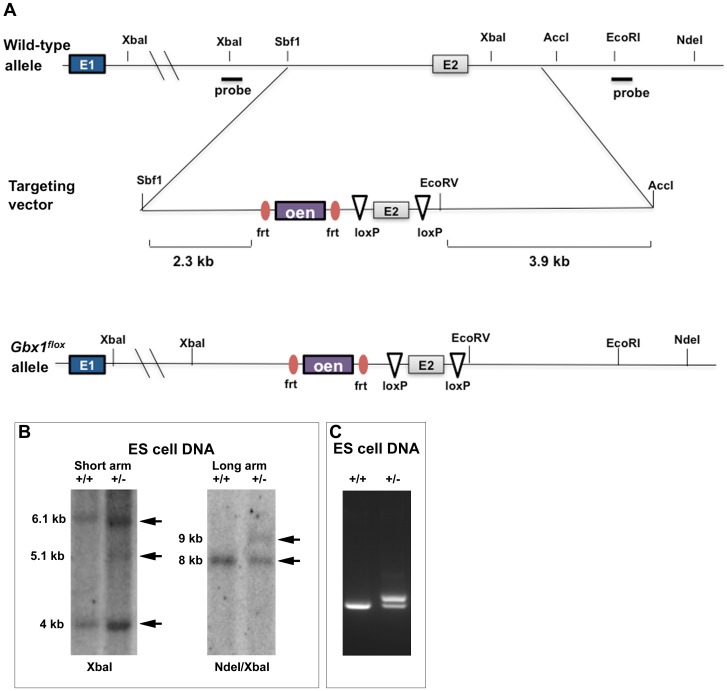
Generation of *Gbx1* mutant mice. (A) For the construction of the conditional *Gbx1-* null, *loxP* sites were inserted flanking exon 2 and a neomycin expression cassette was inserted 5′ to the flanked region as depicted in the schematic. Deletion of exon 2, thereby rendering the endogenous gene null, is mediated by the mating of a mouse containing the targeted allele with a mouse ubiquitously expressing ß-actin Cre. (B) To identify ES cells that properly underwent homologous recombination, the long and short homology arms were screened by Southern Blot of restriction digested genomic DNA. (C) The presence of the WT and targeted alleles in the progeny derived from targeted ES cells were confirmed by PCR of genomic DNA using specific primer sets. Arrow in (A) represents direction of neo transcription; Arrowheads in (A) represent the location of PCR primers used in (C); Gray box indicates exon 2 (E2), which encodes the *Gbx1* homeodomain; Purple box indicates the *neo* cassette flanked by FRT sites (orange circles); White triangles represent the position of *loxP* sites.

**Figure 2:**
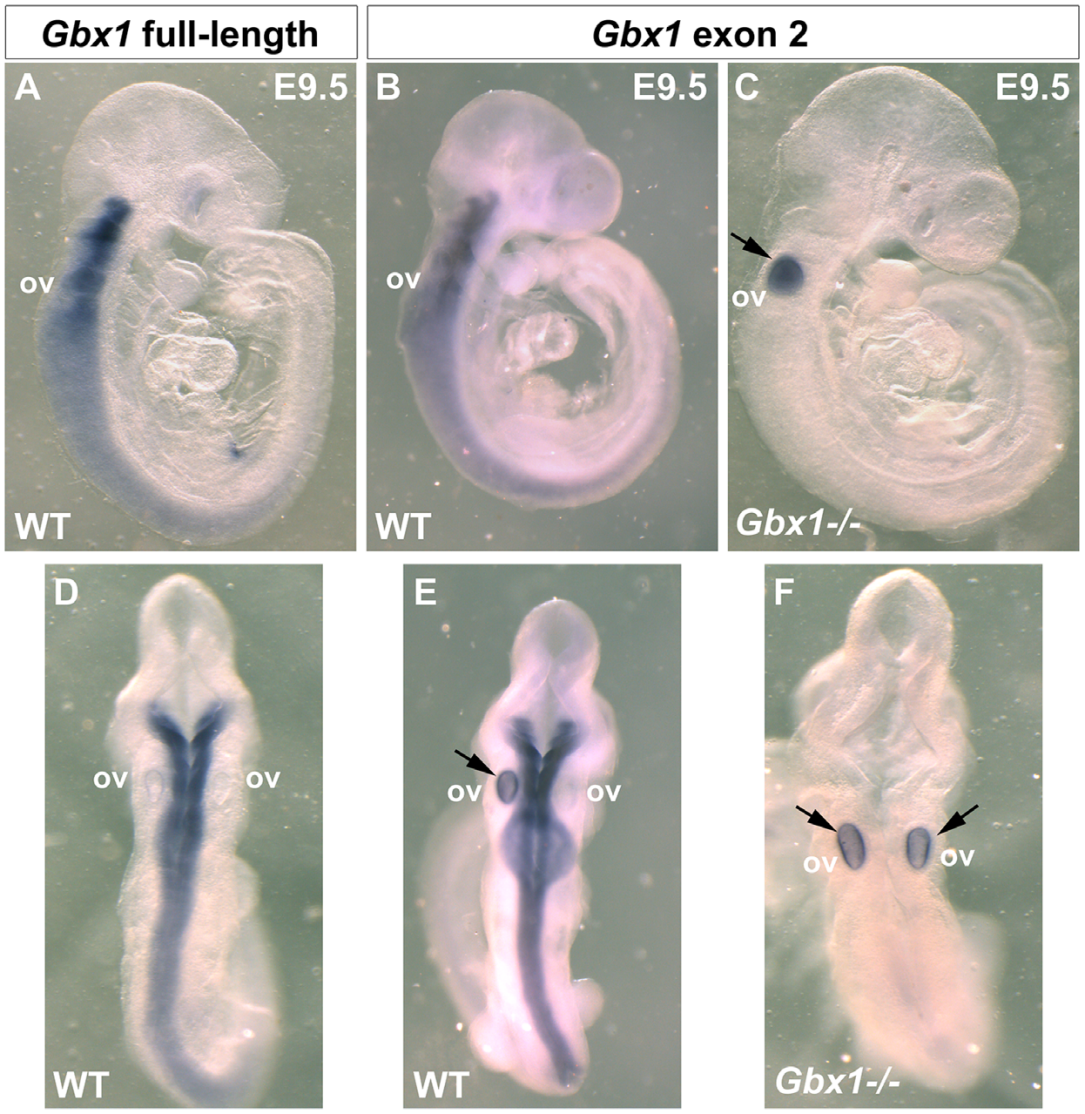
The functional homeodomain of *Gbx1* is deleted in *Gbx1^−/−^* mutants. (A–F) Whole-mount in situ hybridization for *Gbx1* full-length or *Gbx1* exon 2 mRNA at embryonic (E) day 9.5. (A–C) Lateral view, dorsal is to the left. (D–F) Dorsal view. (A, D) *Gbx1* full-length expression in a *Gbx1* WT embryo. Strong *Gbx1* expression detected within the anterior hindbrain with a lessening gradient as expression extends caudally. Expression not detected within the otic vesicles. (B and E) *Gbx1* exon 2 expression in a *Gbx1* WT embryo. Expression of sequence encoding the functional HD of GBX1 recapitulates the pattern detected using the full-length RNA probe. Otic vesicle staining observed within the dorsal view is nonspecific (arrow). (C and F) *Gbx1* exon 2 expression in a *Gbx1^−/−^* embryo. No specific staining observed throughout the entire embryo, demonstrating deletion of the GBX1 functional domain. Otic vesicle staining observed is nonspecific (arrows). ov, otic vesicle.

To study the role of *Gbx1* in development of neural networks, we generated mice carrying a conditional *Gbx1* allele (*Gbx1^flox^*), by flanking exon 2 that contains the sequence encoding the functional DNA-binding homeodomain with *lox*P sequences (Fig. 1A, materials and methods). Accurate recombination of the short and long homology arms into the endogenous *Gbx1* locus of embryonic stem (ES) cells was confirmed through Southern blotting using XbaI and NdeI/XbaI digested genomic DNA, respectively, (Fig. 1B) and PCR (Fig. 1C). Germline transmission of the *Gbx1^flox^* allele was determined by PCR and *Gbx1^flox/+^* heterozygotes were intercrossed to obtain mice homozygous for the *Gbx1^flox/flox^* allele. Unlike *Gbx2^neo/neo^* mice, which die at the day of birth [20], [21], *Gbx1^flox^* homozygotes are viable and do not display any overt behavioral phenotypes. Therefore, to eliminate *Gbx1* function, *Gbx1^flox/flox^* mice were mated with transgenic mice that express a DNA recombinase gene in the early embryo under the control of human *ß-actin* regulatory elements [29]. This resulted in mice with the genotype *Gbx1^−/+^*. *Gbx1^−/+^* heterozygotes were then intercrossed to generate *Gbx1* null mutants (*Gbx1^−/−^*). We examined if mRNA expression in exon 2, which encodes the functional DNA-binding homeodmain is absent in *Gbx1^−/−^* mice. We analyzed wild-type and *Gbx1^−/−^* embryos by in situ hybridization at E 9.5, using *Gbx1* full-length and *Gbx1* exon 2-specific probes. A comparison in wild-type embryos shows that expression of full-length (Fig. 2A and D) and exon 2-specific probes (Fig. 2B and E) are identical. Exon 2 expression is absent in *Gbx1^−/−^* embryos, indicating that the functional DNA-binding domain has been deleted (Fig. 2C and F). The staining observed in the otic vesicle was due to unspecific trapping of the color precipitate (Fig. 2C, E, F). *Gbx1^−/−^* mice are obtained in a ratio in accordance with Mendelian genetics, are fertile, and are as viable through postnatal maturation, as their normal littermate counterparts.

**Figure 3:**
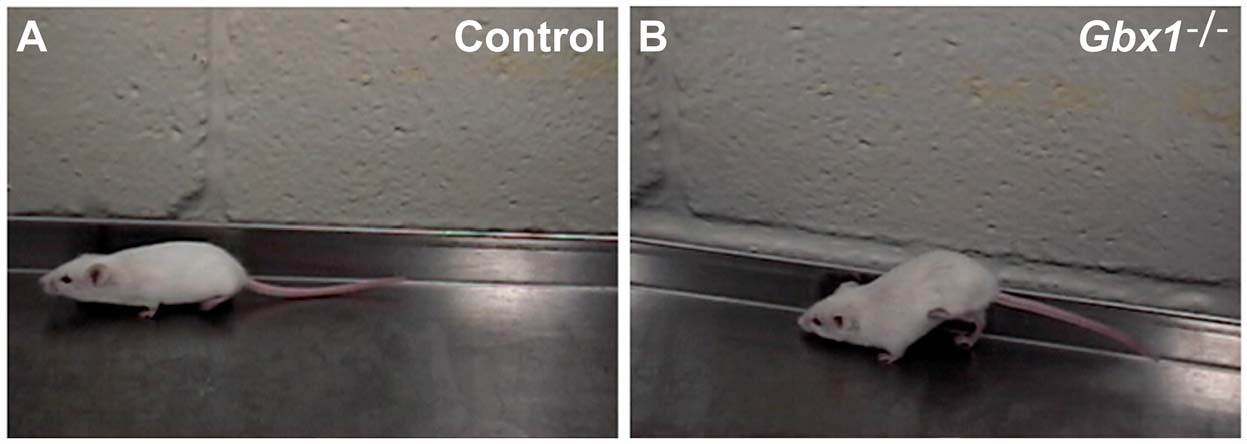
*Gbx1^−/−^* mice display a profound locomotive defect severely affecting hindlimb gait. Photograph depiction of the locomotive phenotype observed in a *Gbx1^−/−^*3-month-old mouse (A) compared to *Gbx1^−/+^* age-matched control (B).

Mice heterozygous for the null mutation displayed no overt behavioral abnormalities. Surprisingly, in *Gbx1^−/−^* mice, we observe a locomotive defect that specifically, and bilaterally, affects hindlimb gait (Fig. 3B, Movie S1). Descriptively, the phenotype is characterized as a prolonged step cycle period with overall increased amplitude of the locomotive rhythm. The abnormal gait is observed as early as P15 and persists at a constant level of severity until death of the animal. While the locomotive defect does not progressively exacerbate in mutant mice, the degree to which the phenotype affects different animals varies, ranging from mild to severe (Movie S1).

### Ventral Spinal Motor Neurons Appear Specified in *Gbx1^−/−^* Embryos

**Figure 4:**
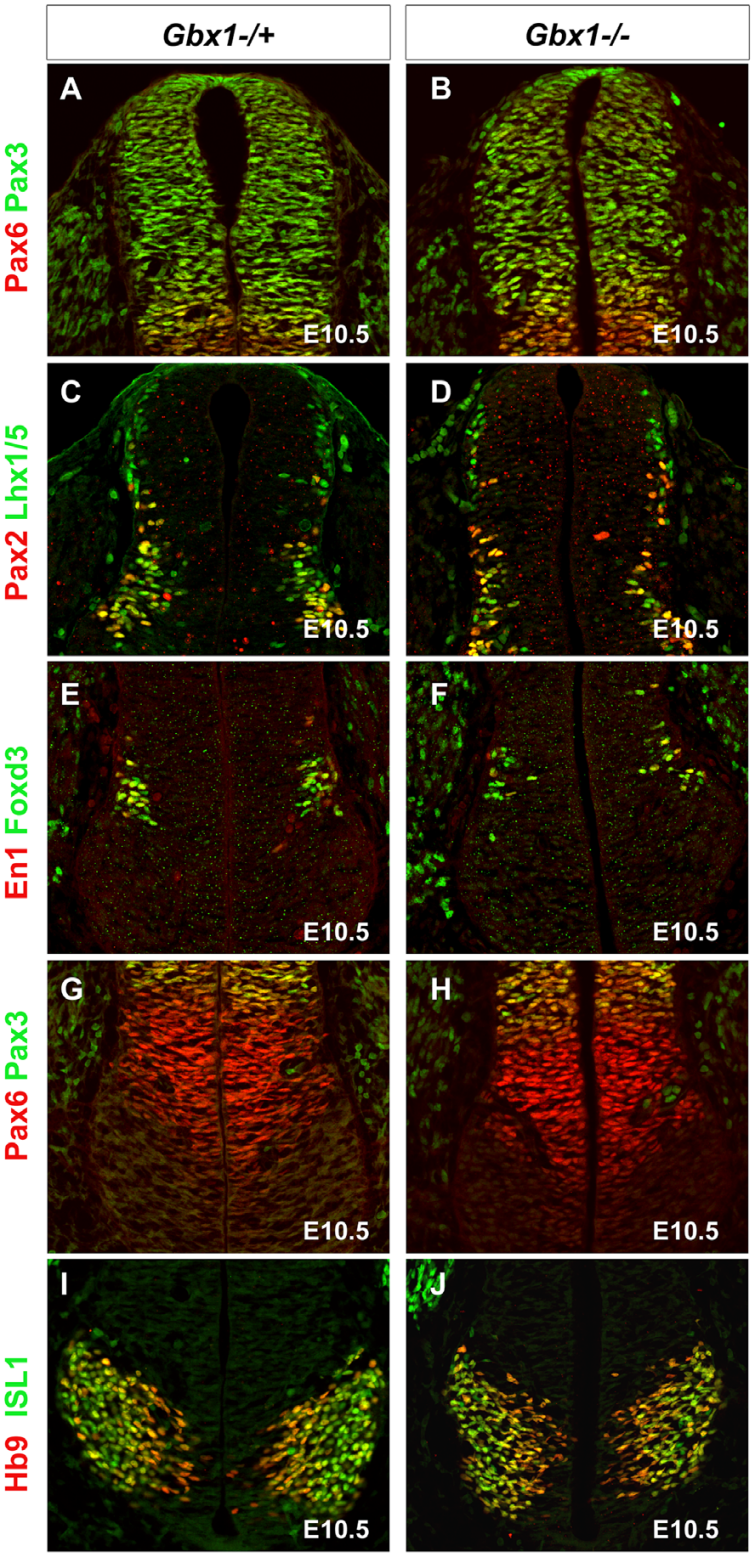
Deletion of *Gbx1* does not affect the patterning of embryonic developmental markers that direct specification of progenitor cell identity within the neural tube. Immunostaining of E10.5 spinal cords to detect localization of proteins required for the acquisition of distinct spinal neuron progenitor populations: Pax6 [dI4-pMN] and Pax3 [dI1-dI6] (A–B and G–H); Pax2 [dI4, dI6-v1] and Lhx1/5 [dI2, dI4, dI6-v1] (C–D); En1 [v1] and Foxd3 [dI2, v1] (E–F); HB9 [pMN] and ISL1 [pMN] (I–J). The expression domains, including the dorsal and ventral boundaries of the various genetically distinct populations of cells do not appear perturbed in *Gbx1^−/−^* mutants (panels in right column), when compared to *Gbx1^−/+^* age matched controls (panels in left column).

The early onset of *Gbx1* expression in the dorsal and ventral ventricular zone of the spinal cord at E9.0–E10.5, suggests a role for *Gbx1* in the specification and generation of defined spinal neuronal subpopulations [26]. We performed a series of immunohistochemical analyses to examine the expression of a panel of molecular markers including, basic helix-loop-helix and homeodomain transcription factors, normally expressed within distinct precursor cell populations throughout the dorsal and ventral spinal cord [15]. Comparison of *Gbx1^−/+^* and *Gbx1^−/−^* embryos at E10.5 did not reveal any apparent differences in expression of these markers (Fig. 4A–J).

The execution of motor response from sensory stimuli is the result of activated MNs within the ventral spinal cord and subsequent transmission of that signal through axonal projections that target muscles in the periphery. The homeobox gene *Hb9*, is expressed at the onset of motor neurogenesis, E9.5, where it is essential for the specification of MN cell fate and is maintained postmitotically as a critical factor for MN differentiation [30]. A key indicator of motor neuron differentiation is the expression of a LIM-homeodomain transcription factor, Islet1 (ISL1), just after exiting the cell cycle which consolidates MN cell fate from pMNs [31]–[33].

**Figure 5:**
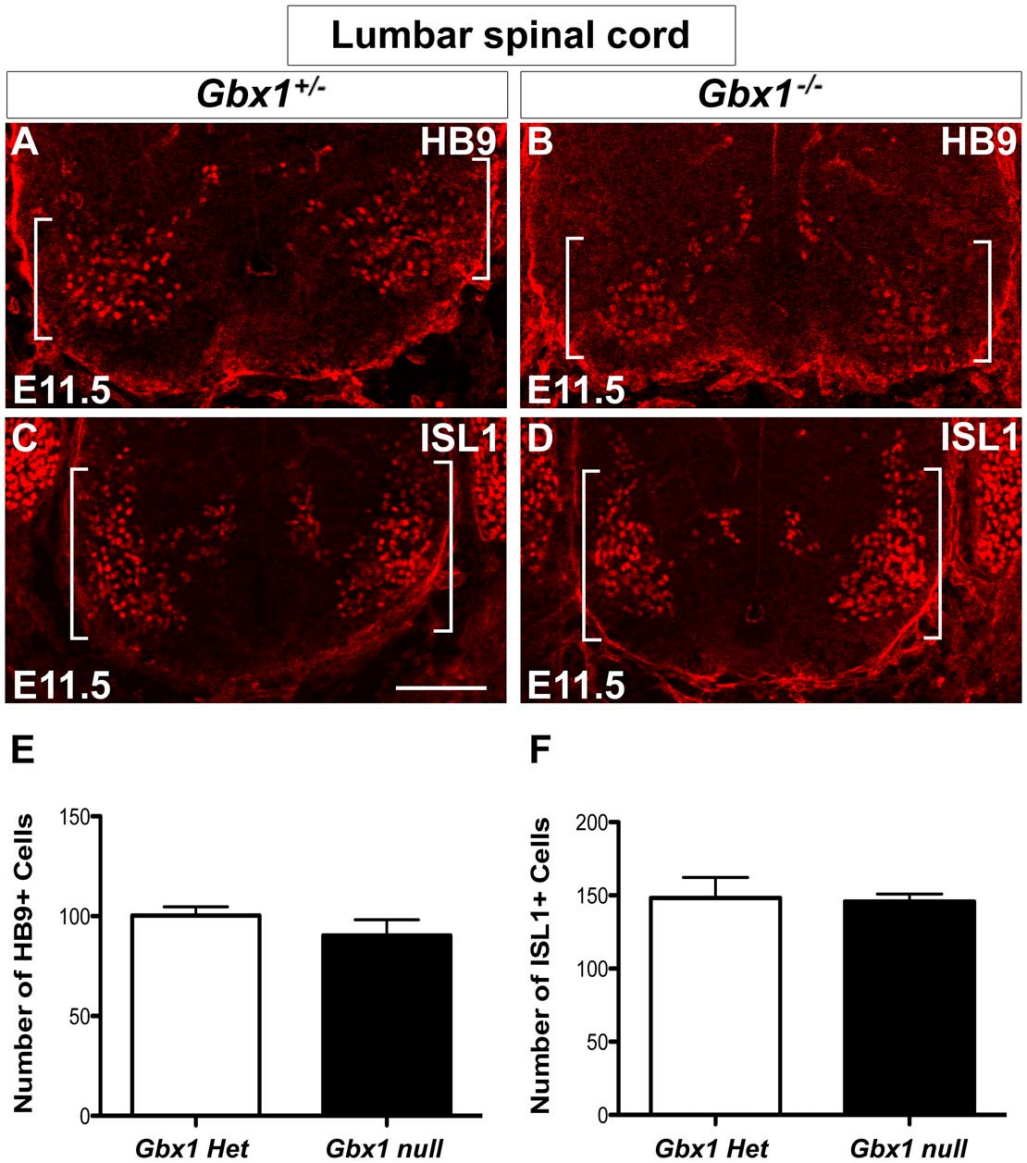
Deletion of *Gbx1* does not affect the generation of ventral spinal cord motor neurons. Immunostaining to detect HB9+ (A–B) and ISL1+ (C–D) motor neurons (indicated by white brackets) at E11.5 in lumber spinal cord sections. Specification of *Gbx1* null motor neurons appears unaffected (B and D) when compared with normal embryos (A and C). Quantification of HB9+ (E) and ISL1+ (F) ventral motor neurons in *Gbx1^−/−^* embryos at E11.5, reveals no significant differences in the number of immunopositive cells when compared to heterozygous littermate controls. Scale bar represents 100 μm. 20X magnification.

To address the defective locomotive phenotype exhibited by *Gbx1^−/−^* mutants, we analyzed the molecular composition of MN populations within the ventral spinal cord using immunohistochemical analyses during several developmental stages of neural generation. At E10.5–E11.5, no significant difference in expression of HB9 (Fig. 5A, B) and ISL1 (Fig. 5C, D) in ventral motor neurons was observed between *Gbx1^−/+^* and *Gbx1^−/−^* mice (Figure 4I and J). By E11.5 greater than (85%) of the total pMN population has differentiated into motor neurons [7]. Therefore, these results indicate that the pre-mitotic pMNs that gave rise to the observed postmitotic HB9^+^ and ISL^+^ ventral spinal MNs at these stages were likely properly specified. Taken together, these data suggest no apparent disruption in the generation of the neuronal population which functions to relay motor output signals to the periphery.

### *Gbx1^−/−^* Embryos Display Abnormal Projection of Proprioceptive Sensory Axons and a Decrease in Peripherin^+^ Ventral Motor Neurons

**Figure 6:**
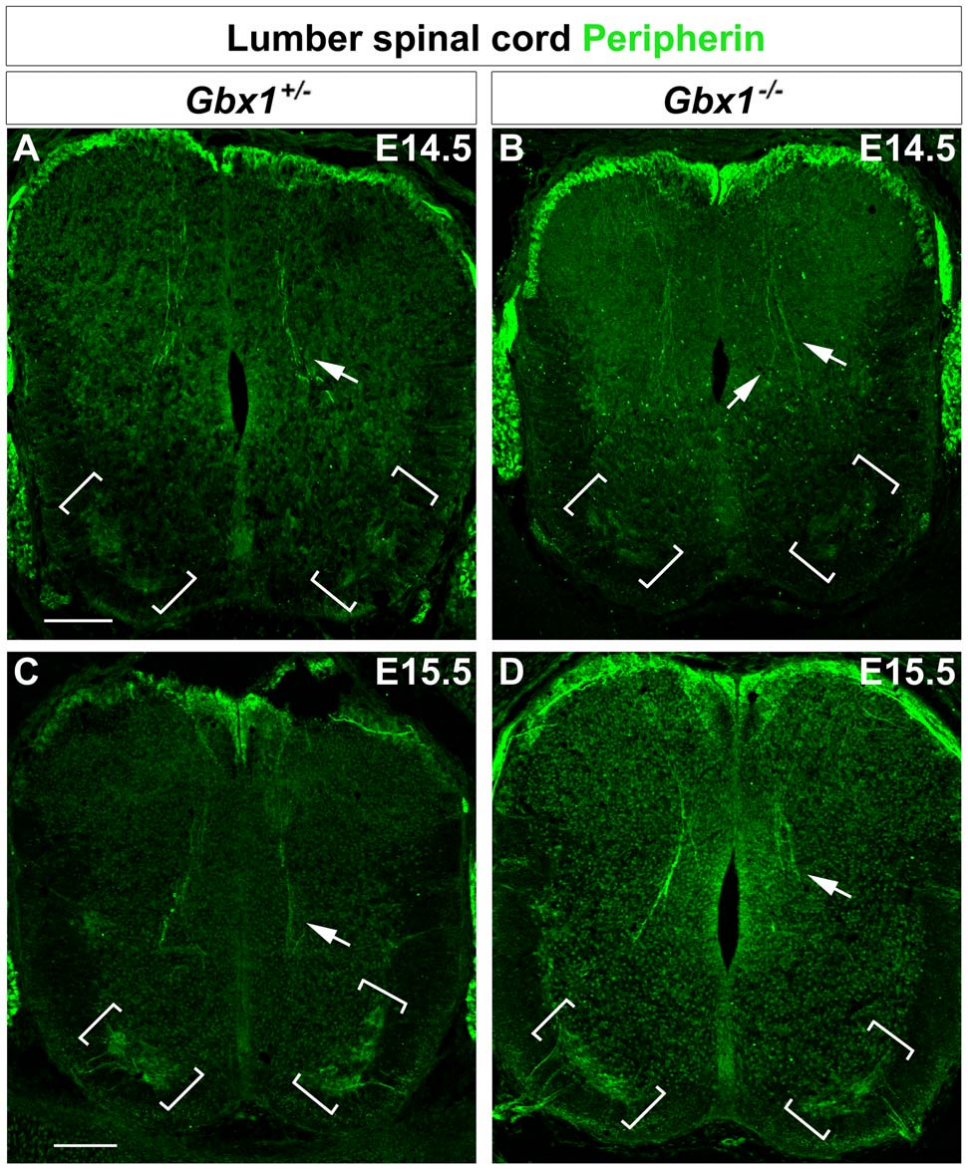
*Gbx1^−/−^* embryos display abnormal projection of proprioceptive afferents and decrease in peripherin+ ventral motor neurons. Peripherin immunolabeling in lumbar spinal cord sections at E14.5 and E15.5. Arrows indicate proprioceptive afferents extending into the spinal cord. Control mice show normal projection of afferents into the intermediate spinal cord (A) and ventral termination zone (C). Many of the proprioceptive afferents fail to project into the ventral spinal cord of *Gbx1^−/−^* mice (B and D). *Gbx1^−/−^* embryos also show a marked decrease in the expression of peripherin+ ventral motor neurons (brackets) in (B and D) when compared to control mice (A and C). Scale bars represent 100 μm. 10X magnification.

Since there was no major difference in molecular composition or gross morphological assembly observed of the motor neuron populations in our *Gbx1* null mutant when compared to the normal control at E11.5, we sought to examine the neural systems that synchronize somatosensory stimuli and that might address the locomotive phenotype. Peripherin is a type III intermediate filament protein that is abundantly expressed within developing spinal motor neurons and primary proprioceptive afferent axonal projections in the dorsal spinal cord [34], [35]. To examine anatomical constituents of the proprioceptive modality, we analyzed the expression of peripherin by immunohistochemistry in transverse sections of the lumbar spinal cord during mid-embyronic stages. At E14.5, peripherin expression is observed along the length of primary sensory afferents projecting into the dorsal spinal cord through the dorsal root entry zone (DREZ) and marks a subset of ventral motor neurons, in *Gbx1^−/+^* embryos (Fig. 6A). This expression profile is altered in *Gbx1^−/−^* embryos at E14.5. While peripherin expression persists within the axonal afferents they appear disorganized with several axons extending ectopically (compare white arrow in Fig. 6B and 6A). In addition, we observed a considerable decrease in peripherin expression within the subset of ventrally marked motor neurons (brackets in Fig. 6B). By E15.5, the perturbed assembly of the proprioceptive sensory axon afferents become significantly disarrayed in mutant embryos, displaying a premature termination of ingrowth to their intended target zone and interneural connection, the Ia interneurons (compare white arrow in Fig. 6D and 6C). Additionally, further examination of peripherin expression within the ventral motor neurons of control and *Gbx1^−/−^* embryos demonstrates that the significant decrease in expression persists through E15.5 (Fig. 6D).

**Figure 7:**
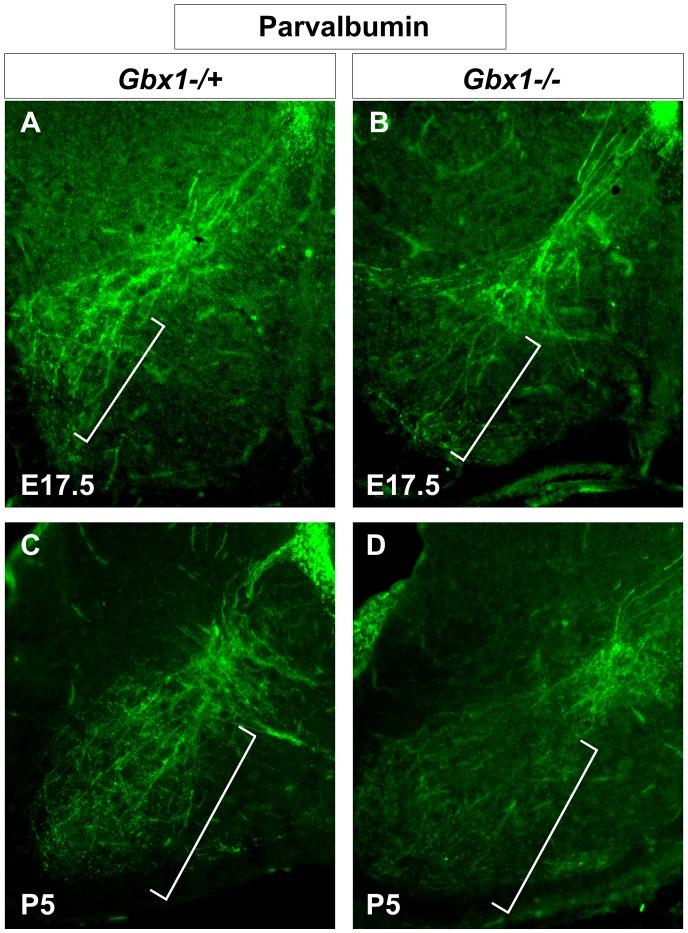
*Gbx1^−/−^* embryos continue to display abnormal projection of proprioceptive afferents at late embryonic and early postnatal stages in development. Parvalbumin immunolabeling in transverse lumbar spinal cord sections at E17.5 and P5. Brackets indicate the innervation of proprioceptive afferents into the intermediate and ventral spinal cord where they are destined to make synaptic connections with their interneuron or motor neuron targets, respectively. Control mice show normal projection of afferents to their intermediate and ventral termination zones (A and C). Many of the proprioceptive afferents fail to fully project to their ventral termination zones in the spinal cord of *Gbx1^−/−^* mice (B and D), while maintaining their proper termination in the intermediate spinal cord. 10X magnification.

The data shown above through mid-embryonic stages, strongly suggest that components of the proprioceptive system are disrupted in spinal cords of *Gbx1* null embryos during early stages of motor circuit assembly. However, it is important to note that synaptogenesis and the ontogeny of motor development are not completed until early postnatal stages. To determine if change in the projection of proprioceptive sensory afferent axons persist during late-embryonic and postnatal stages, we assessed the axonal expression of parvalbumin (PV) a marker of proprioceptive neurons. At E17.5 the projection of proprioceptive afferents into the ventral termination zone characteristic of group Ia afferents was nearly absent in *Gbx1^−/−^* mutants when compared to controls (Fig. 7A and B). The first phase of postnatal maturation and synaptogenesis occurs between postnatal day (P) 0 and P8 [36]. Importantly, we show that the dramatic reduction of proprioceptive afferents into the ventral horn of *Gbx1* mutants persist through P5, a stage when synaptogenesis and proliferation of proprioceptive synapses occurs [36] (Fig. 7C and D). Taken together, these data demonstrate that essential components of the proprioceptive system are disrupted in *Gbx1* null embryos, which may serve as a contributing factor to the locomotive phenotype.

### *Gbx1^−/−^* Embryos Display a Decrease in Both ISL1^+^ and ISL1^+^/Peripherin^+^ Ventral MNs

**Figure 8:**
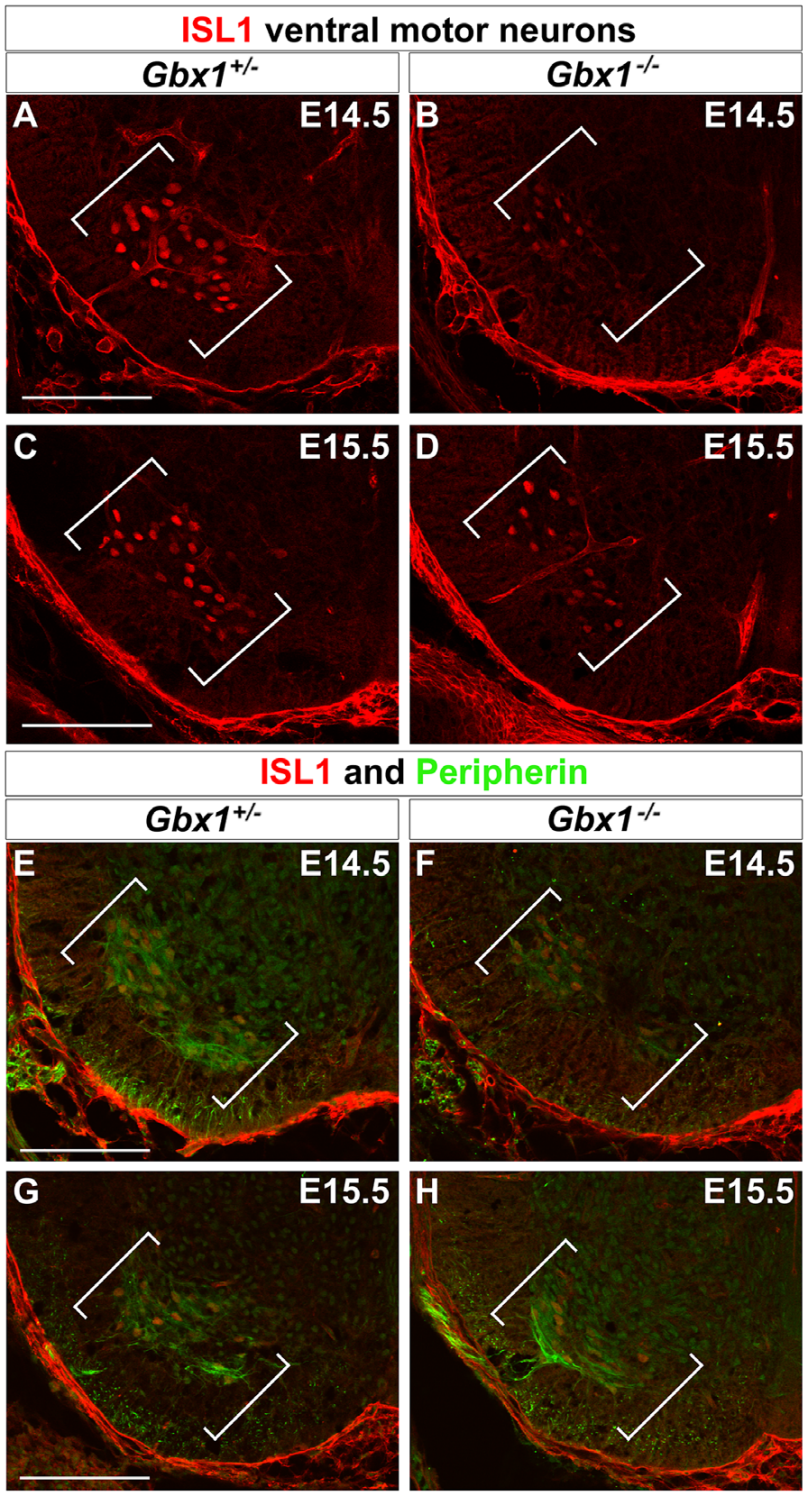
ISL1+ and ISL1+/peripherin+ co-expressing motor neurons are reduced in *Gbx1−/−* ventral spinal cord. Immunohistochemical analysis for ISL1 (A–D) and ISL1+/peripherin+ co-expressing cells (E–H) in lumbar spinal cord sections at E14.5 and E15.5. Expression of ISL1+ motor neurons (A and C) and ISL1+/peripherin+ co-expressing cells (E and G) in the ventral spinal cord of control embryos. *Gbx1^−/−^* embryos show a significant reduction in the number of ISL1 ventral motor neurons at E14.5 and E15.5 (B and D) and motor neurons coexpressing ISL1/peripherin (F and H). Scale bars represent 100 μm. 20X magnification.

**Figure 9:**
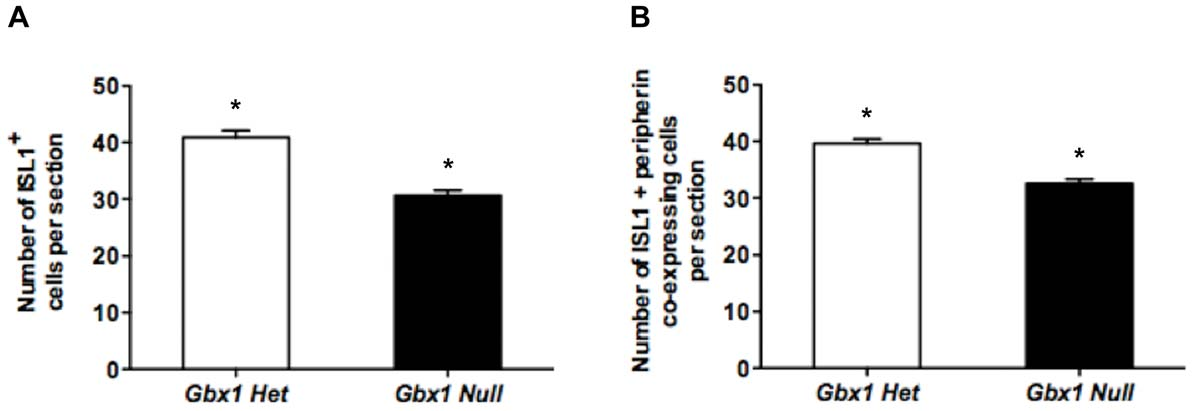
Quantification of ISL1+ and ISL1+/peripherin+ ventral motor neurons in *Gbx1^−/−^* embryos. Quantification of ISL1 expressing motor neurons (A) and ISL1/peripherin coexpressing motor neurons (B) in the lumbar ventral spinal cord of E14.5 and E15.5 embryos. Each bar represents the average from 10 sections (n = 4) for null and (n = 4) for heterozygotes; *P<0.0001.

Since we observed a decrease in the population of peripherin-immunoreactive ventral motor neurons at a later stage of spinal neural development, we chose to revisit and further examine those populations for ISL1, at comparable developmental stages (E14.5–15.5). At both stages examined, there is a dramatic reduction in ISL1-immunoreactive ventral motor neurons in *Gbx1^−/−^* embryos when compared to control embryos (Fig. 8A–D). Quantified measurement of the ISL1 immunohistochemical assay reveals a significant reduction in the total number of ISL1^+^ cells in the ventral spinal cord (Figure 9A; *P*<0.0001).

This observation prompted us to investigate whether the subset of motor neurons which lose peripherin immunoreactivity between E14.5–E15.5 are the same subset of motor neurons that lose ISL1 immunoreactivity at the same developmental stage. Thus, we examined spinal cord sections of control and *Gbx1* null embryos co-stained with ISL1 and peripherin. The results show co-localization of ISL1 and peripherin in a subset of ventral motor neurons, in mutant and control embryos (Figure 8E–H). We observed a comparable loss in the expression of ISL1 and peripherin co-immunopositive motor neurons in the vMN pool to that of our single staining analyses. This suggests that the reduced population of ventral cell bodies observed in our previous immunohistochemical assays is the same subset of ISL1^+^ and peripherin^+^ motor neurons. This conclusion is supported by quantification of the ISL1+ peripherin immunopositive cell bodies, which affirms a marked attenuation of the ventral motor neurons in embryos lacking functional *Gbx1* (Figure 9B; *P*<0.0001). Furthermore, we show that the significant reduction in ventral ISL1+ cells persists through E17.5 in *Gbx1^−/−^* embryos (Fig. S1). Together, these results indicate that *Gbx1^−/−^* embryos suffer from a severe reduction in the number of vMNs expressing the hallmark motor neuron marker, ISL1, and which also express the axonal growth factor peripherin [37], likely contributing to the locomotive phenotype.

### The Population of Proprioceptive Sensory Neuron Cell Bodies within the Dorsal Root Ganglion Remains Unaffected in *Gbx1^−/−^* Mutants

**Figure 10:**
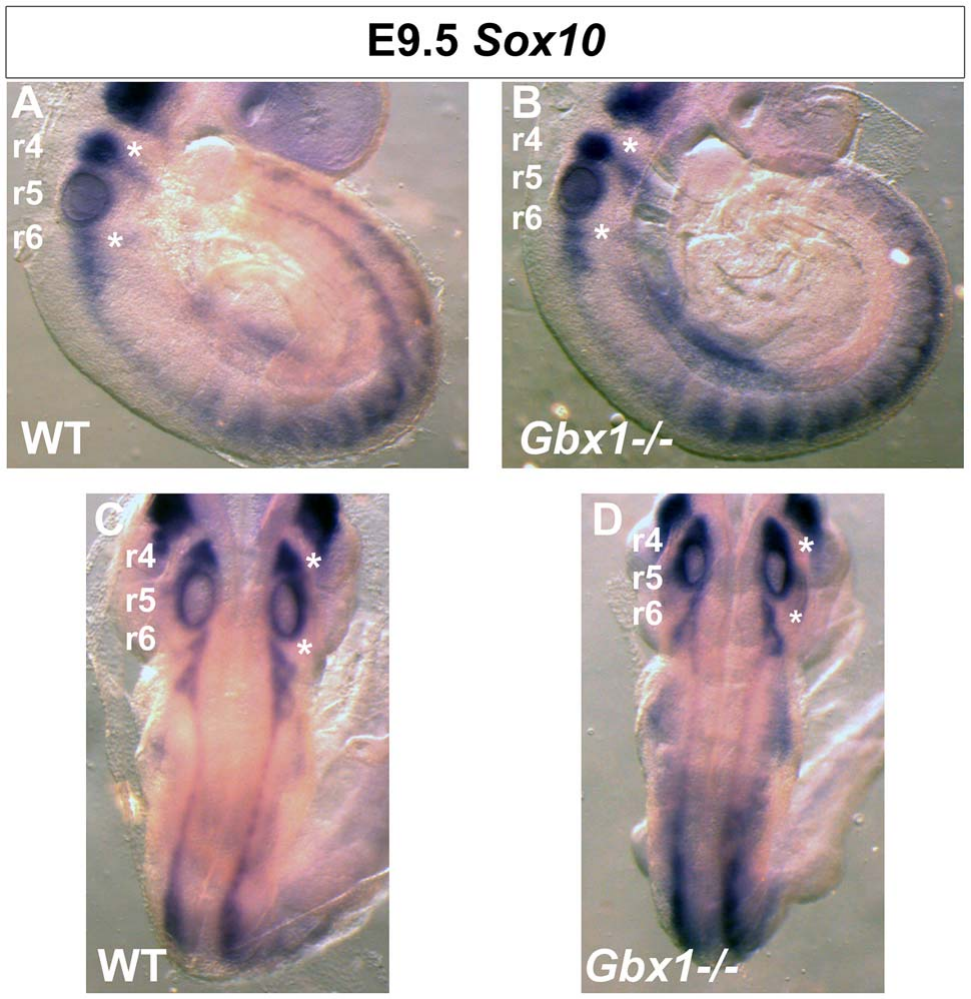
Expression analysis of *Sox10* in *Gbx1^−/−^* embryos. (A–D) Whole-mount in situ hybridization for *Sox10* expression at E9.5. (A–B) Lateral view, dorsal is to the left. (C–D) Dorsal view. *Sox10* expression detected in the r4/r6 hindbrain neural crest streams (asterisks) and within the dorsal root ganglia in the trunk adjacent to the developing spinal cord, is largely unaffected in a *Gbx1* ***^−/−^*** mutant embryo (B) compared to a littermate control embryo (A). r, rhombomere.

**Figure 11:**
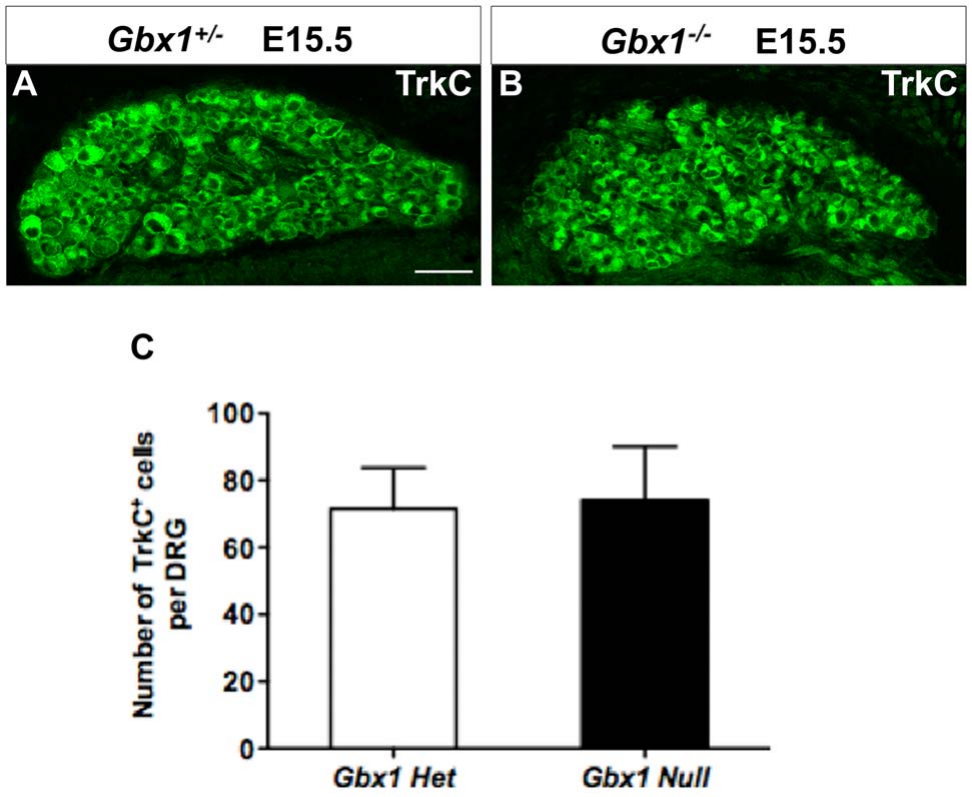
The number of proprioceptive neurons in the DRG is not affected in *Gbx1−/−* **mice.** The distribution of proprioceptive neurons marked by the expression of TrkC antibody in the DRG of embryos at E15.5 (A and B). The proprioceptive neurons show no difference in the expression between the *Gbx1^−/−^* (B) and control embryos (A). Quantification of TrkC expressing neurons in the DRG (C). Each bar represents the average of six DRGs from (n = 3) heterozygous and (n = 3) for *Gbx1^−/−^* embryos; *P*<0.8107, non-significant; Scale bar represent 100 μm. 20× magnification.

Currently, there are no studies identifying a role for *Gbx1* in NC cell development. Recent studies in *Xenopus*, however, indicate that *Gbx2* is the earliest factor in the genetic cascade of NC induction regulated by Wnt signaling [38]. The perturbation of constituents that mediate the internal transmission of the proprioceptive modality in *Gbx1^−/−^* embryos prompted us to analyze the NC-derived components that initiate proprioceptive perception. We have previously shown that *Gbx1* is expressed in the r4 and r6 NC streams of the anterior hindbrain at E9.0 [26]. To determine if NC cells are specified and migrate correctly in *Gbx1^−/−^* embryos, we examined the expression of the NC marker *Sox10* in wild-type and *Gbx1^−/−^* embryos at E9.5. We observed no apparent differences in *Sox10* expression between wild-type and mutant embryos in the r4/r6 streams or trunk DRG at this stage (Fig. 10A–D). The NC-derived cell bodies functionally responsible for integrating spatial orientation of the organism reside in the DRG. Thus, we examined the DRG for the neurotrophic factor TrkC, which is the molecular marker for proprioceptive sensory neuron cell bodies. TrkC^+^ neurons are generated during the first two, of three waves of sensory neuron genesis occurring between E9.5–E14.5 [39]. Furthermore, the diversification of sensory subtypes generated during the first and second waves to those that functionally implement proprioceptive stimuli occurs at E14.5, through the co-expression of the RUNX family transcription factor *Runx3* and TrkC [40]. Our immunohistochemical analysis showed no overt differences to the morphology of the TrkC^+^ pool of neurons between our mutant and control animals (Fig. 11A, B). Quantification of the total number of individual TrkC^+^ cell bodies reinforced the notion that the proprioceptive sensory neuron pool is unaffected in *Gbx1^−/−^* mutant embryos (Fig. 11C). This data provides evidence that the population that defines the origin of the proprioceptive modality is properly established in early stages of development in mutant embryos. This implies that disruption to downstream elements of the proprioceptive system is likely the source of molecular and anatomical manipulation that causes hindrance to the neural network that facilitates normal locomotion in *Gbx1^−/−^* mice.

## Discussion

*Gbx1* is dynamically expressed during embryogenesis, particularly within the CNS. In this study, we have focused our analysis on the functional role(s) *Gbx1* plays in the developing nervous system. By producing mice homozygous for a *Gbx1* loss-of-function allele, we demonstrate that *Gbx1* function is a key regulatory component in assembly of neuronal circuitry controlling normal locomotion. In contrast to mice homozygous for the *Gbx2^−/−^* allele, *Gbx1* mutants are viable. However, consistent with *Gbx2* mutant embryos, *Gbx1* ^−/−^ embryos display severe developmental defects impacting CNS organization and function. Our data show a disruption in the growth of proprioceptive afferents towards the intermediate zone and ventral termination zone in spinal cords of E14.5–E15.5 *Gbx1* mutant embryos when compared to normal embryos. In addition, we show a significant loss of ISL1^+^ and ISL1^+^/peripherin^+^ co-expressing ventral motor neurons. These abnormalities are detected at E14.5 and become more apparent at E15.5. Furthermore, we show that the reduction of proprioceptive afferent projection into the ventral horn of *Gbx1* mutants persist through P5, when synaptogenesis of proprioceptive synapses occurs. Collectively, the results from our analysis of *Gbx1^−/−^* mutants from E14.5–P5 provide strong evidence that *Gbx1* function is required in aspects key to the formation, interconnection and maintenance of sensorimotor circuits in the spinal cord. In addition, the data provide new genetic insights towards the elucidation of the molecular mechanisms underlying somatosensory-related gait disorders.

### Loss of *Gbx1* Function Impacts the Late Stages of Sensorimotor Circuit Development

During development, sensory neurons of the DRG send axons to the CNS and to sensory receptors in the periphery [18], [41], [42]. Several classes of transcription factors have been implicated in the early developmental processes of specification, patterning, and selection of early axonal trajectories of different classes of sensory neurons [17], [18], [41]–[44]. For example, the generation of all DRG sensory neurons requires the combinatorial expression of basic helix-loop-helix proteins Neurogenin 1 and Neurogenin 2 [39]. More recent studies show that *Runx3*, a member of the Runt family of transcription factors, regulates development and survival of proprioceptive afferents. In addition, *Runx3*-deficient mice display severe motor discoordination and limb ataxia [40], [41], [45]. Unlike mice harboring mutations for the above stated transcription factors, we detected no change in the total number of TrkA^+^, TrkB^+^ or TrkC^+^ DRG sensory neurons in *Gbx1^−/−^* embryos when compared to normal embryos (Fig. 11, data not shown). Thus it is unlikely that *Gbx1* expression has a significant role in the establishment or diversification of DRG sensory neurons.

DRG sensory axons and motor axons reach their peripheral target areas prior to the entry of DRG sensory afferents into to the spinal cord [46], [47]. Depending on their sensory modality, sensory neurons of the DRG send axons to superficial layers of the dorsal horn, (nociception and thermoception), or the deep layers of the dorsal horn, lateral horn and ventral horn, (mechanoreceptive and proprioceptive) [11], [12], [18]. The early onset of *Gbx1* expression in the dorsal and ventral ventricular zone of the spinal cord at E9.0–E10.5, is consistent with the specification and generation of defined spinal neuronal subpopulations, suggesting a possible role for *Gbx1* in their integration into neuronal circuits [26]. In contrast to that theory, our immunohistochemical analyses of *Gbx1^−/−^* embryos at E10.5 did not uncover any abnormal expression of a panel of molecular markers including, basic helix-loop-helix and homeodomain transcription factors, normally expressed within the distinct precursor cell populations throughout the dorsal spinal cord (Fig. 4). Collectively, these data strongly suggest that *Gbx1* expression does not impact the early steps underlying the formation of sensorimotor circuits. Nevertheless, recent studies have shown that *Gbx1* expression in the spinal cord is dynamic and becomes restricted to the dorsal mantle zones at E12.5. Immunohistochemical analyses of wild-type embryonic and adult spinal cords demonstrate that *Gbx1* is expressed in late-born LBX1*^+^* (class B) neurons from E12.5–E16.5, distinguishes a distinct subpoplation of GABAergic dorsal spinal neurons and could function in the late steps of spinal circuit assembly [26], [27].

Proprioceptive neurons begin to project afferents into the dorsal spinal cord at E14.0, before cutaneous afferents terminate in the dorsal horn, and into the deep dorsal horn by E15.0 [46]. Establishment of connections between Ia afferents and ISL1^+^ motor neurons in the ventral horn begins at E15.5 and continues until P8 [44], [46]. In *Gbx1^−/−^* embryos, projection of proprioceptive afferents into the intermediate and ventral spinal cord terminates prematurely (Figs. 6 and 7). As a result, these mutant mice lack many of the direct synaptic connections normally formed with motor neurons in the ventral termination zone, correlating well to the severe hindlimb motor discoordination. Interestingly, the late neuronal and behavioral phenotypes observed in *Gbx1* null mutants resembles mild forms of the motor control defects seen in mutant mice with major alterations in proprioceptive neuronal circuitry [40], [45], [48]. For example, *Er81*, a member of the ETS transcription factor family is expressed in both developing motor neurons and proprioceptive sensory neurons. Results from studies of *Er81* mutant mice exhibit a failed formation of a discrete termination zone between Ia proprioceptive afferents and motor neurons in the ventral spinal cord. However, specification of motor neurons and induction of muscle spindles in *Er81* mutant mice occurs normally. Furthermore, it is interesting to note that similar to *Gbx1^−/−^* mice, *Er81* mutants display severely uncoordinated limb movements [44].

In addition to projection of proprioceptive afferents into the intermediate and ventral spinal cord, our data demonstrate a requirement of *Gbx1* for normal patterning of ISL1^+^ ventral motor neurons, another key component of sensorimotor circuits in vertebrates. Motor neurons are within the earliest born neurons of the ventral spinal cord [49], [50]. The first postmitotic motor neurons in the mouse spinal cord are detected at E9–E9.5, and the generation of motor neurons is complete by E11.0 [49]. We and others have shown that *Gbx1* is expressed in the prospective spinal cord at E9.0 and in the ventral ventricular zone at an anatomical level that coincides with motor neuron progenitor cells by E10.5 [26], [51]. Consistent with our analysis of dorsal spinal cord precursor cells of *Gbx1^−/−^* embryos, our immunohistochemical analyses at E10.5 did not uncover any abnormal expression of ISL1, HB9 or a panel of transcription factors expressed within the distinct precursor cell subtypes throughout the ventral spinal cord (Fig. 4). Furthermore, no apparent difference in the total number of postmitotic ISL1^+^ and HB9^+^ ventral motor neurons was observed in *Gbx1* mutant embryos at E11.5 when compared to normal controls (Fig. 5).

Intriguingly, we observed a marked reduction in the total number of ISL1^+^ and ISL1^+^/peripherin^+^ motor neurons at later stages in development, E14.5–15.5, in *Gbx1^−/−^* embryos compared to normal embryos. These results further support a role for *Gbx1* in establishment of sensorimotor connections. However, our results raise two distinct issues concerning the mechanism underlying the loss of motor neurons at this late stage of development of *Gbx1* mutant embryos. First, a considerable amount of neuronal loss occurs amongst differentiated, post-migrational neurons that are in the process of establishing connections between afferents and target neurons [52]. We have shown that premature termination of proprioceptive afferents occurs in the intermediate zone of *Gbx1^−/−^* spinal cords from E14.5–P5 mutants. As a consequence, functional connections with motor neurons in the ventral target zone may not be made, resulting in a loss of motor neurons through programmed cell death [53]. In contrast, recent studies in mice have demonstrated that reduced levels of Islet protein favors the generation of V2a interneurons at the expense of motor neuron formation [54]. In support of this notion, cell-fate conversion of motor neurons occurs in zebrafish upon knockdown of *isl1* and *isl2* [55]. Our previous *in situ* hybridization analyses show that *Gbx1* expression coincides with a population of motor neurons in the ventral spinal cord at E10.5 [26]. In this study we show that inactivation of *Gbx1* does not result in a failure to specify ISL1^+^ motor neurons. Yet, we observed a significant decrease in the total number of motor neurons in *Gbx1* mutant embryos. While our study does not address this question directly, it presents the hypothesis that *Gbx1* can play a role in the maintenance of ISLl1 expression in a subset of motor neurons, preventing their conversion into V2a interneurons. However, this possibility remains to be determined empirically.

### Abnormal Locomotion in *Gbx1* Mutants

Our analysis of *Gbx1^−/−^* mice has revealed a novel role for Gbx transcription factors in regulating the assembly of sensorimotor circuits and motor behavior. Unlike *Gbx2* mutant mice, *Gbx1* mutants display a striking gait disorder, which specifically affects the hindlimbs. Since *Gbx2^−/−^* mice do not survive beyond birth, we cannot determine the manifestation of a gait disorder. However, *Gbx2* mutants do display cranial nerve V motor neuron and motor control defects during embryogenesis that severely impact hindbrain development and the ability to suckle [20], [24]. In addition, a recent lineage-tracing study using *Gbx2^CreER-ires-eGFP^* mice has demonstrated a requirement for *Gbx2* expression in early progenitor cells of the neural tube (E8.5) for normal development and patterning of ventral motor neurons in the spinal cord to occur [25]. It is also intriguing that *Gbx2* mutant embryos develop with severe inner ear defects affecting vestibular function, which could contribute to impairment of movement and coordination [56]. Our examination of *Gbx1* mutant mice did not reveal any apparent musculoskeletal or peripheral nervous system defects. And, since *Gbx1^−/−^* mice do not display any abnormal head movements or circling behavior, it is highly unlikely that the phenotype is a result of impaired vestibular function [57]. While our data do not rule out a possible requirement for *Gbx1* expression in regions outside of the spinal cord for normal locomotion, we did not observe changes in other components of the major systems that govern posture and locomotion. Moreover, while *Gbx1* is expressed in the medial ganglionic eminence, which contributes to the formation of the basal ganglia, expression has not been detected in other major components of the motor system outside of the spinal cord, such as the, brainstem, or cerebellum [26].

Movement disorders are caused by a variety of neurological conditions, which manifest into a broad clinical spectrum that includes dystonia, ataxia and gait disorders. Nevertheless, all movement disorders share common features in neural circuits which impair the planning, control or execution of movement [58]. One of the simplest and best understood neuronal circuits in the vertebrate CNS is the spinal monosynaptic stretch reflex circuit, in which connections are formed between a sensory unit and an effecter unit [59]. The precise coordination of movement by this circuit is carried out by connections formed between two main classes of neurons, proprioceptive Ia sensory neurons and ventral spinal motor neurons. Therefore, it is very intriguing that *Gbx1* directly impacts both proprioceptive afferent projection and ventral motor neuron development in the spinal cord. Furthermore, the function of *Gbx1* parallels several transcription factors that control the establishment of connections within the spinal monosynaptic stretch reflex circuit [42]. Group Ia afferents innervate muscle spindles in the periphery and form direct connections with ventral motor neurons in this circuit. We have shown a marked reduction of the group Ia proprioceptive afferents in *Gbx1* mutants. Whereas the group Ib afferents, which project to the intermediate spinal cord and do not make synaptic contact with motor neurons appear normal in *Gbx1* mutants [59]. In summary, our studies revealed a novel role for *Gbx1* in regulating key components involved in the integration of sensorimotor circuitry affecting motor behavior. A challenge now is to further define the mechanisms impacted by a loss of *Gbx1*. Future investigations should be conducted to identify and analyze the direct molecular targets of GBX1. Insight into these factors will provide greater understanding of transcriptional control of the distinct subpopulations of motor and sensory neurons by *Gbx1*.

## Materials and Methods

### Ethics Statement

The work performed in this manuscript is in compliance with the University of Missouri Office of Animal Care Quality Assurance (ACQA) under the protocol number 6479. No IRB approval is needed. The mice were housed and handled in accordance with the University of Missouri Animal Care and Use Committee (ACUC) guidelines. CO_2_ (100%) asphyxiation followed by cervical dislocation was performed to euthanize adult mice. For embryos, immersion in 4% paraformaldehyde was used for embryos E14 or younger. Chilling followed by decapitation was used for embryos older than E14. The invasive procedures used to harvest embryos were only performed following euthanasia.

### Generation of *Gbx1^−/−^* Mice

Mice carrying the mutant null allele for the *Gbx1* gene were generated through homologous recombination of a targeting construct engineered to allow excision of the functional DNA-binding homeodomain. From a bacterial artificial chromosome containing the full-length *Gbx1* genomic sequence, we isolated 14.4 kb of *Gbx1* DNA. Insertion of a *frt*-flanked *neomycin* (*neo*) resistance cassette into the intronic sequence upstream of exon 2 conferred positive selection by G418. *loxP* sequences inserted 5′- and 3′- to exon 2, which contains the functional DNA-binding motif, facilitates recognition by the *Cre* DNA recombinase enzyme and mediates excision of the floxed sequence. ES cells electroporated with the targeting construct were screened by Southern blot analysis of XbaI and NdeI/XbaI restriction digested DNA to identify homologous recombination of the short arm and long arm, respectively.

We generated a mouse line carrying the *Gbx1^flox^* allele by the injection of homologous recombinant ES cells into 129 blastocysts and subsequent mating of the resulting chimeric males to C57BL6 females to obtain germ-line transmission of the targeted allele. Mice carrying the *Gbx1^flox^* allele develop normally, and are reproductively competent. In order to generate the nonfunctional null allele for *Gbx1*, *Gbx1^flox^* homozygous mice were crossed to transgenic mice expressing the *Cre* DNA recombinase under the control of the ubiquitous *ß-actin* promoter, [29] resulting in mice lacking exon 2 (*Gbx1^−/−^*). *Gbx1^−/−^* mice are viable and phenotypically indistinguishable from their littermates at birth. However, by postnatal (P) day 15, *Gbx1^−/−^* mice show a severe locomotor defect affecting hindlimb locomotion.

### Genotype Analysis

Genotyping was achieved by use of PCR using genomic DNA prepared from ES cells, embryonic tissue or adult tail biopsies. To identify the *Gbx1^flox^* allele through PCR, a 5′ forward primer (5′GTTTGCTGTGCGCAGCCAGCA3′) located within exon 2 and a 3′ reverse primer (5′CCTCAGGAATCCACTTCTGCT3′) that anneals immediately downstream of the second *loxP* site, yields a 300 bp product corresponding to the floxed allele. The *Gbx1^−/−^* allele was detected using a 5′ forward primer (5′CGTCAAGAAGGCGATAGAAGG3′) contained within the *neo* cassette and the same 3′ reverse primer (5′CCTCAGGAATCCACTTCTGCT3′) used to detect the floxed allele. PCR analysis was performed under the following parameters: 1) 94°C for 3 minutes, 2) 94°C for 30 seconds, 3) 55°C for 30 seconds and 4) 72°C for 3 minutes (steps 2–4 were repeated for 30 cycles).

### Immunohistochemistry

For immunohistochemistry analyses, *Gbx1^−/−^* and control embryos were dissected and subsequently fixed with 4% paraformaldehyde (PFA) in 1X phosphate-buffered saline (PBS) for 2 hours at 4°C, washed 3 times in 1X PBS for 1 hour, equilibrated with 25% sucrose overnight at 4°C and embedded in opimal temperature tissue (OCT) (Tissue Teck) for cryosectioning. Transverse, serial 20 μm cryosections were made along the length of the spinal cord. Sections were washed with 0.1% Triton X-100 in 1X PBS (PBST), blocked with 1X PBS containing 10% lamb serum, 1% bovine serum albumin, and 0.25% Triton X-100 for 90 minutes and incubated with the appropriate primary antibodies in blocking solution at 4°C overnight. The following day, sections are washed briefly with PBST and incubated with the appropriate fluorescently conjugated secondary antibodies in blocking solution at 4°C overnight. The following primary and secondary antibodies we used at the given dilution: mouse monoclonal anti-islet1(1∶100, DSHB), mouse anti-HB9 (1∶100; DSHB), rabbit anti-peripherin (1∶200; Millipore), rabbit polyclonal anti-TrkC (1∶200; Santa Cruz Biotech), rabbit anti-parvalbumin (1∶500, Calbiochem), goat anti-rabbit AlexaFluor 488 (1∶500; Invitrogen), and goat anti-mouse AlexaFluor 488 or 568 (1∶500; Invitrogen). Stained sections were dehydrated in serial dilutions of ethanol in 1×PBS and mounted using DPX mounting media or glycerol mounting media containing DAPI.

### In Situ Hybridization

Whole-mount RNA in situ hybridizations were performed as previously described [26]. To demonstrate that exon 2, which contains the sequence encoding the functional DNA-binding homeodomain (HD) of GBX1, is successfully deleted in the *Gbx1* null mutants, a 588 bp cDNA fragment consisting of the *Gbx1*-HD sequence was amplified from genomic DNA using PCR and cloned in the pBluescript KS(−) vector. *Sox10* anti-sense RNA probe was provided by A. Chandresekhar and construct was engineered by P. Trainor’s lab. For in situ hybridizations digoxigenin (Roche Molecular Biochemicals) labeled probes were used.

### Microscopy

Analysis of immunostained spinal cords and DRG sections were examined and photographed using the Zeiss 510 META confocal microscope under 10X and 20X ocular magnification. Identical parameters were used consistently for each experiment.

### Statistical Analysis

Statistical analyses of the experiments were performed using the Graphpad Prism software. The unpaired student’s t-test algorithm were applied to the data sets and are represented as mean+SEM. Samples were considered statistically significant having a value of *P*<0.05.

## Supporting Information

Figure S1 **Reduction of ISL1+ motor neurons in** ***Gbx1^−/−^*** **ventral spinal cord persists at a late stage in embryonic development.** Immunohistochemical analysis for ISL1+ cells in lumbar spinal cord sections at E17.5 Expression of ISL1+ motor neurons (A) in the ventral spinal cord of control embryos. *Gbx1^−/−^* embryos show a qualitatively observable significant reduction in the number of ISL1+ ventral motor neurons. 20X magnification.
(TIF)

Movie S1 **Abnormal hindlimb gait in Gbx1 mutant mice.** The video shows the gait of one normal (first white mouse) and two mutant mice (second white mouse, first agouti mouse).
(MOV)
