# Supplementary figures and images for: Characterization of the Gbx1−/− Mouse Mutant: A Requirement for Gbx1 in Normal Locomotion and Sensorimotor Circuit Development
Source: PLoS One. 2013 Feb 13;8(2):e56214. doi: 10.1371/journal.pone.0056214 (PMC3572027; doi:10.1371/journal.pone.0056214)

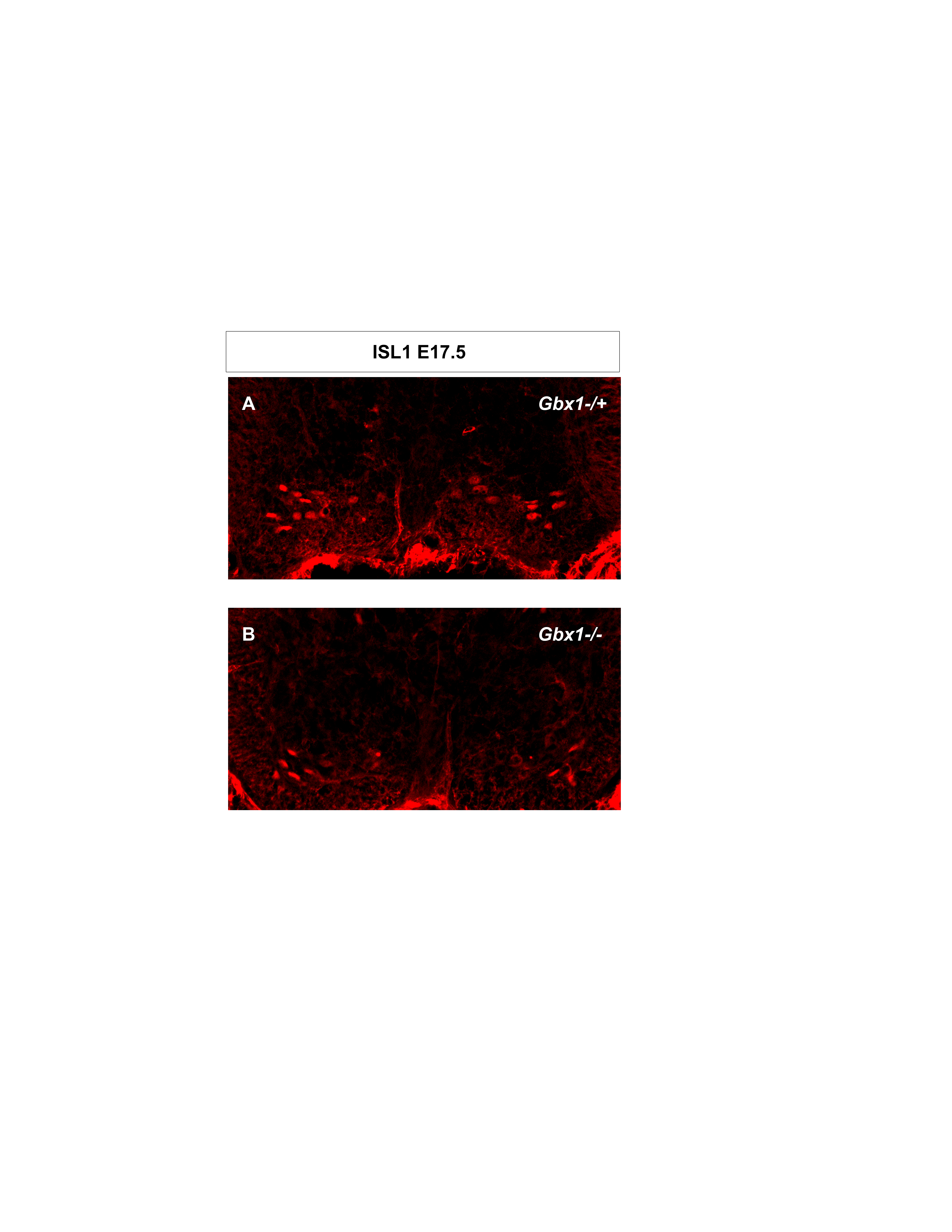

Supplement: Figure S1 — Reduction of ISL1+ motor neurons in Gbx1−/− ventral spinal cord persists at a late stage in embryonic development. Immunohistochemical analysis for ISL1+ cells in lumbar spinal cord sections at E17.5 Expression of ISL1+ motor neurons (A) in the ventral spinal cord of control embryos. Gbx1−/− embryos show a qualitatively observable significant reduction in the number of ISL1+ ventral motor neurons. 20X magnification. (TIF) [file pone.0056214.s001.tif]
